# Supplementary material for: Oxford Nanopore R10.4 long-read sequencing enables the generation of near-finished bacterial genomes from pure cultures and metagenomes without short-read or reference polishing
Source: Nat Methods. 2022 Jul 4;19(7):823–6. doi: 10.1038/s41592-022-01539-7 (PMC9262707; doi:10.1038/s41592-022-01539-7)
Supplement: Supplementary file 1 — Supplementary Note, Supplementary Figs. 1–10 and Supplementary Tables 1–4. [file 41592_2022_1539_MOESM1_ESM.pdf]

nature portfolio

https://doi.org/10.1038/s41592-022-01539-7

Supplementary information

Oxford Nanopore R10.4 long-read sequencing enables the generation of near-finished bacterial genomes from pure cultures and metagenomes without short-read or reference polishing

In the format provided by the authors and unedited

Supplementary information for

Oxford Nanopore R10.4 long-read sequencing enables near-finished bacterial genomes from pure cultures and metagenomes without short-read or reference polishing

Mantas Sereika[1*], Rasmus Hansen Kirkegaard[1,2*], Søren Michael Karst[1], Thomas Yssing Michaelsen[1], Emil Aarre Sørensen[1], Rasmus Dam Wollenberg[3] and Mads Albertsen[1**]

[1]Center for microbial communities, Aalborg University, Denmark

[2]Joint Microbiome Facility, University of Vienna, Austria

[3]DNASense ApS, Denmark

*These authors contributed equally to the paper

**Corresponding author ma@bio.aau.dk

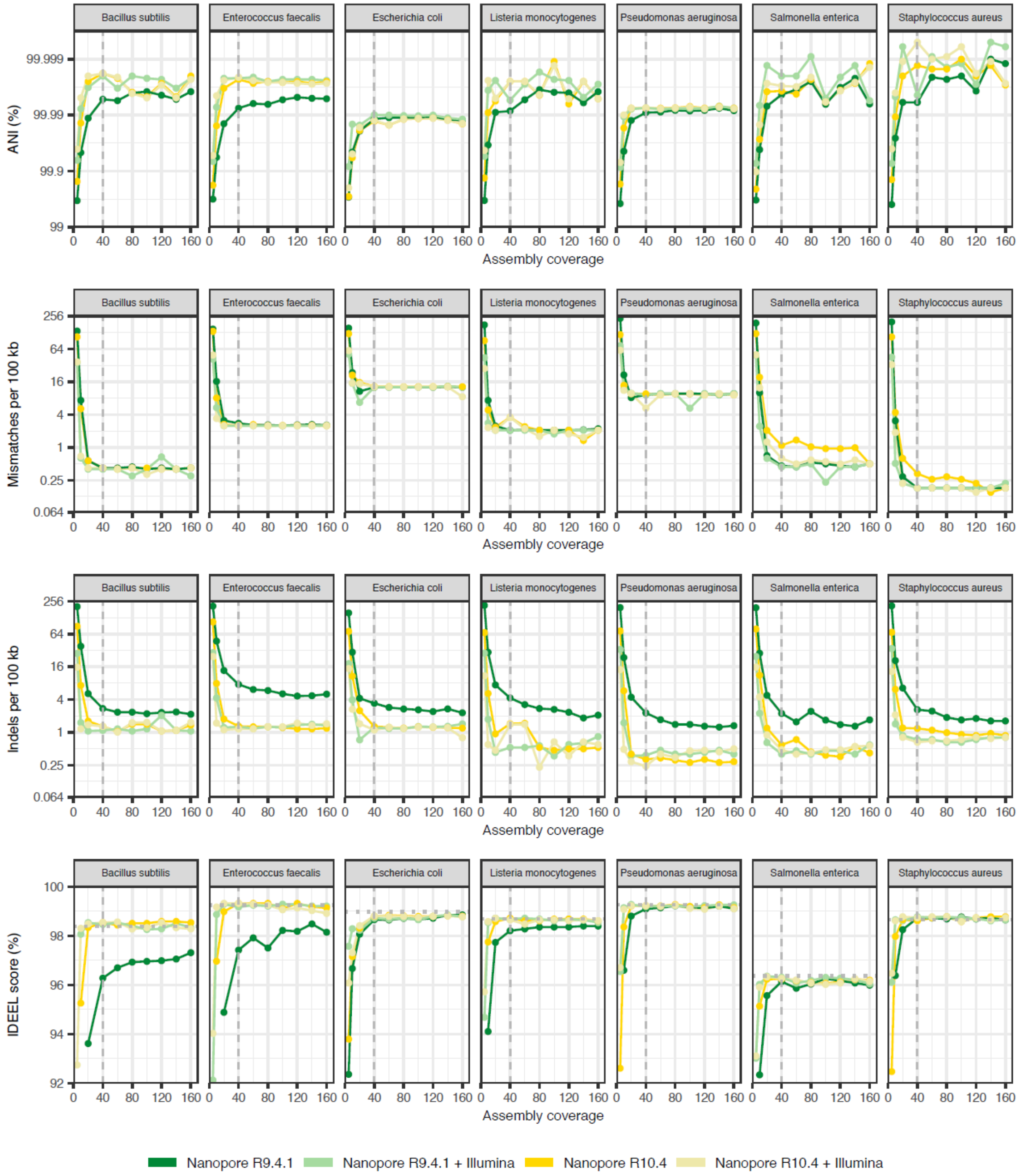

Figure S1: Assembly quality metrics for the ZYMO Mock HMW DNA.

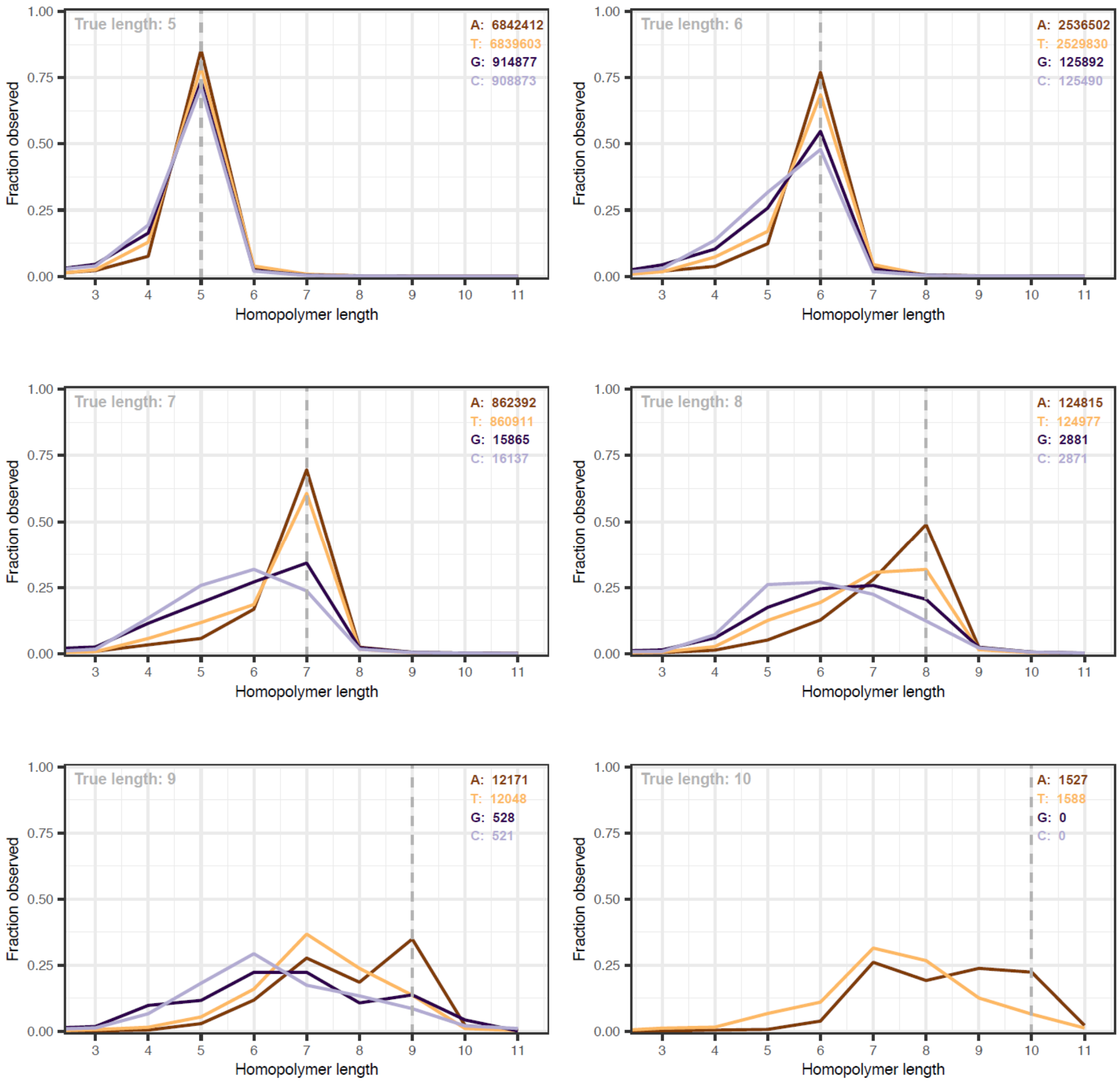

Figure S2: Counterr homopolymer plots for Nanopore R9.4.1 read data of the Zymo HMW mock. Reads for each Zymo mock species, subsetted to a coverage of 160, were used for the analysis.

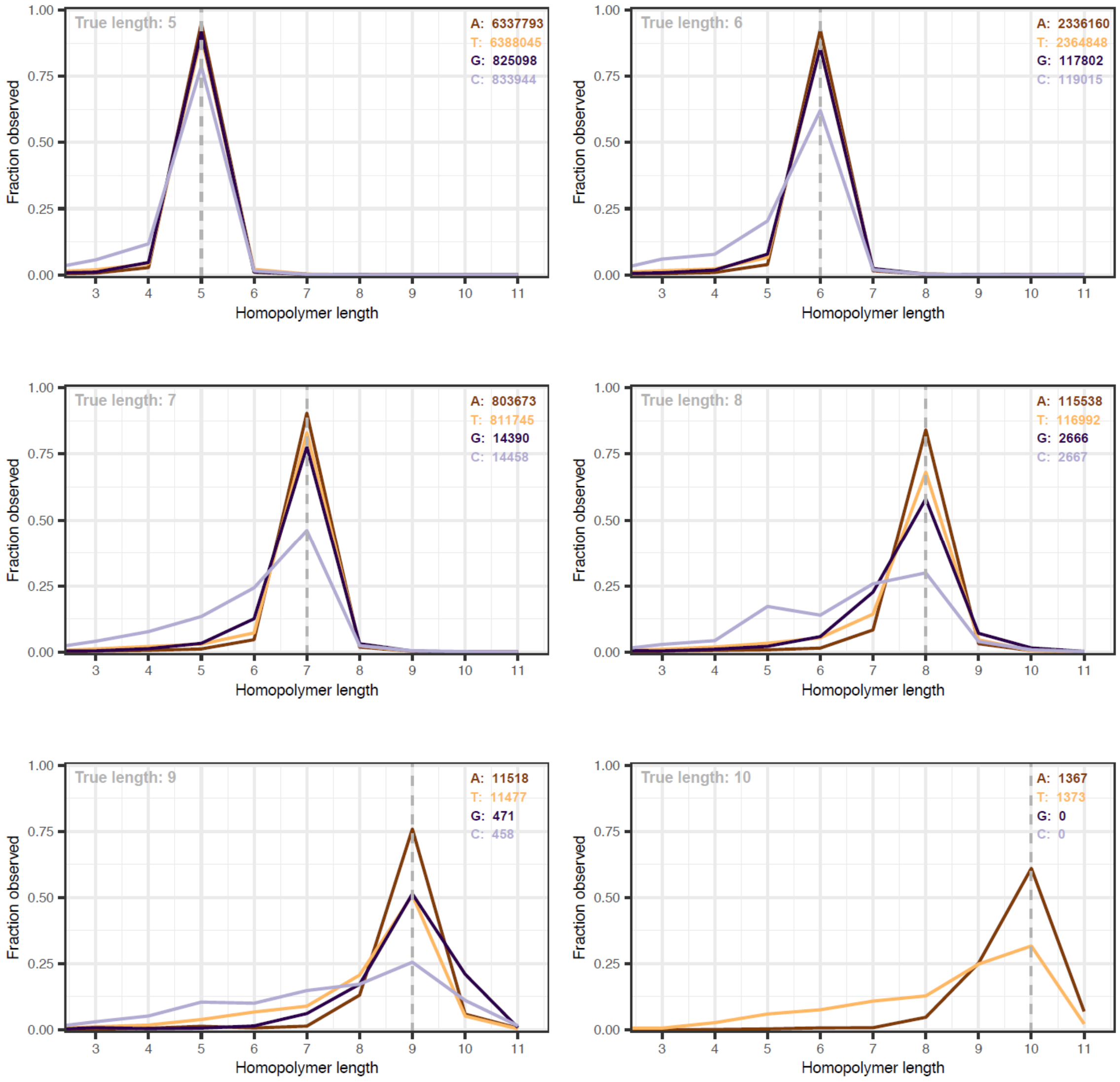

Figure S3: Counterr homopolymer plots for Nanopore R10.4 read data of the Zymo HMW mock. Reads for each Zymo mock species, subsetted to a coverage of 160, were used for the analysis.

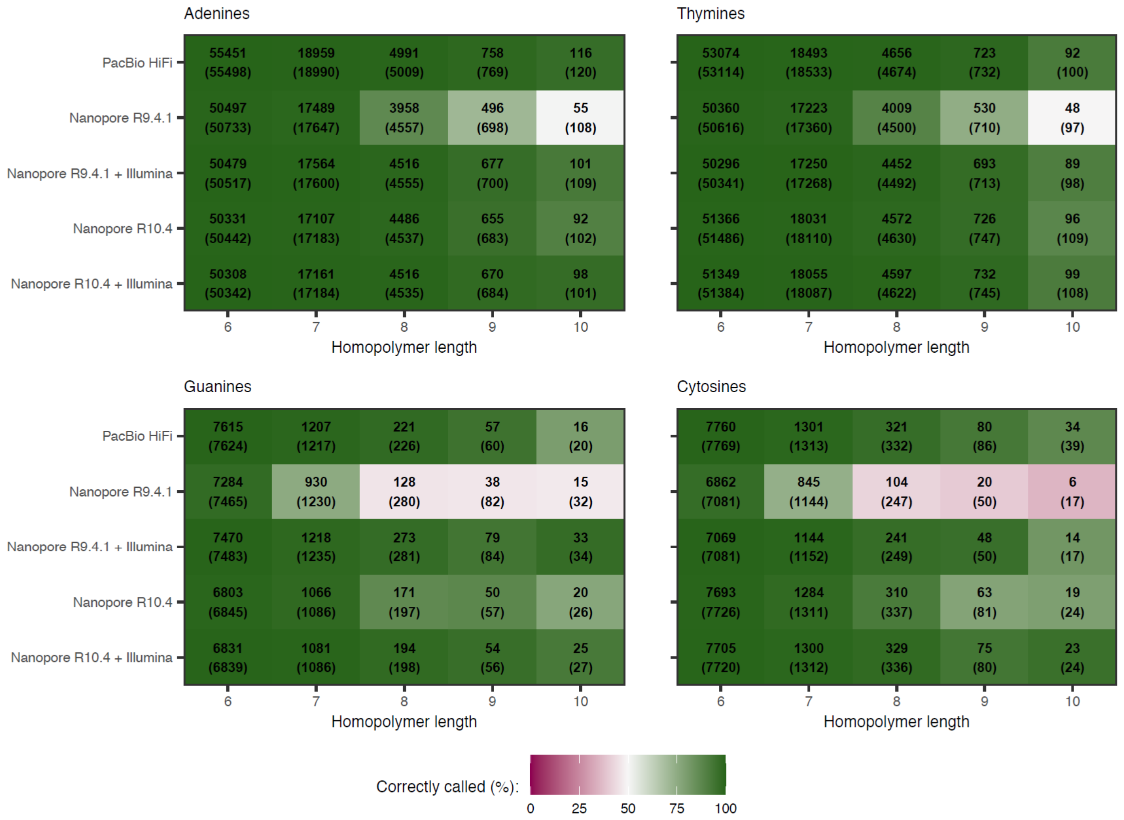

Figure S4: Homopolymer calling estimates in metagenomes (consensus sequences) from different sequencing platforms, acquired from comparison to Illumina-polished PacBio HiFi metagenome assembly. Values in the heatmap show observed homopolymer counts estimated to be called correctly at a given sequence length. The total count of homopolymers (called correctly and incorrectly) are in brackets. Only the contigs for bins that were clustered together between long-read sequencing platforms and featured a coverage higher than 10 were used to generate values for the plot.

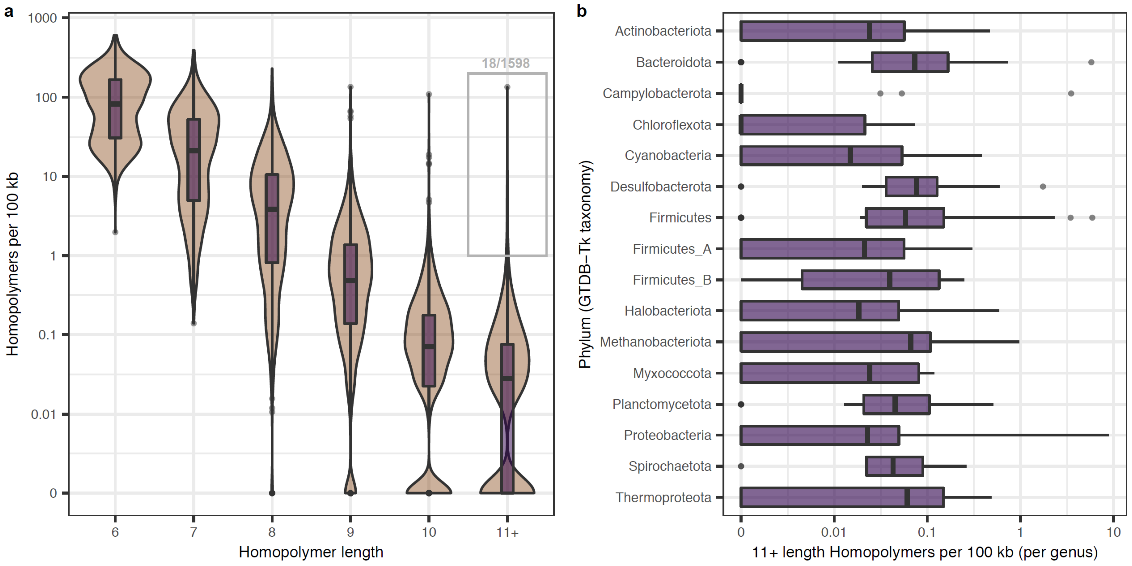

Figure S5: Homopolymer counting results for RefSeq genome database. **a)** Homopolymer counts by different lengths in bacterial and archaeal genomes. To avoid database overrepresentation, we subsampled the data to select a genome per unique genus (n=1,598). **b)** Long homopolymer (+11 length) counts for different phyla, using subsampled genomes, which feature GTDB-Tk taxonomy (n=1,043). Each phyla consists of a minimum of 10 genomes. The line within the boxplot denotes the median, while the hinges correspond to the 75th and 25th percentiles. The upper whisker extends up to 1.5 times the interquartile range (IQR), whereas the lower whisker extends down to the smallest value of 1.5 times the IQR. Points beyond the whiskers are plotted separately.

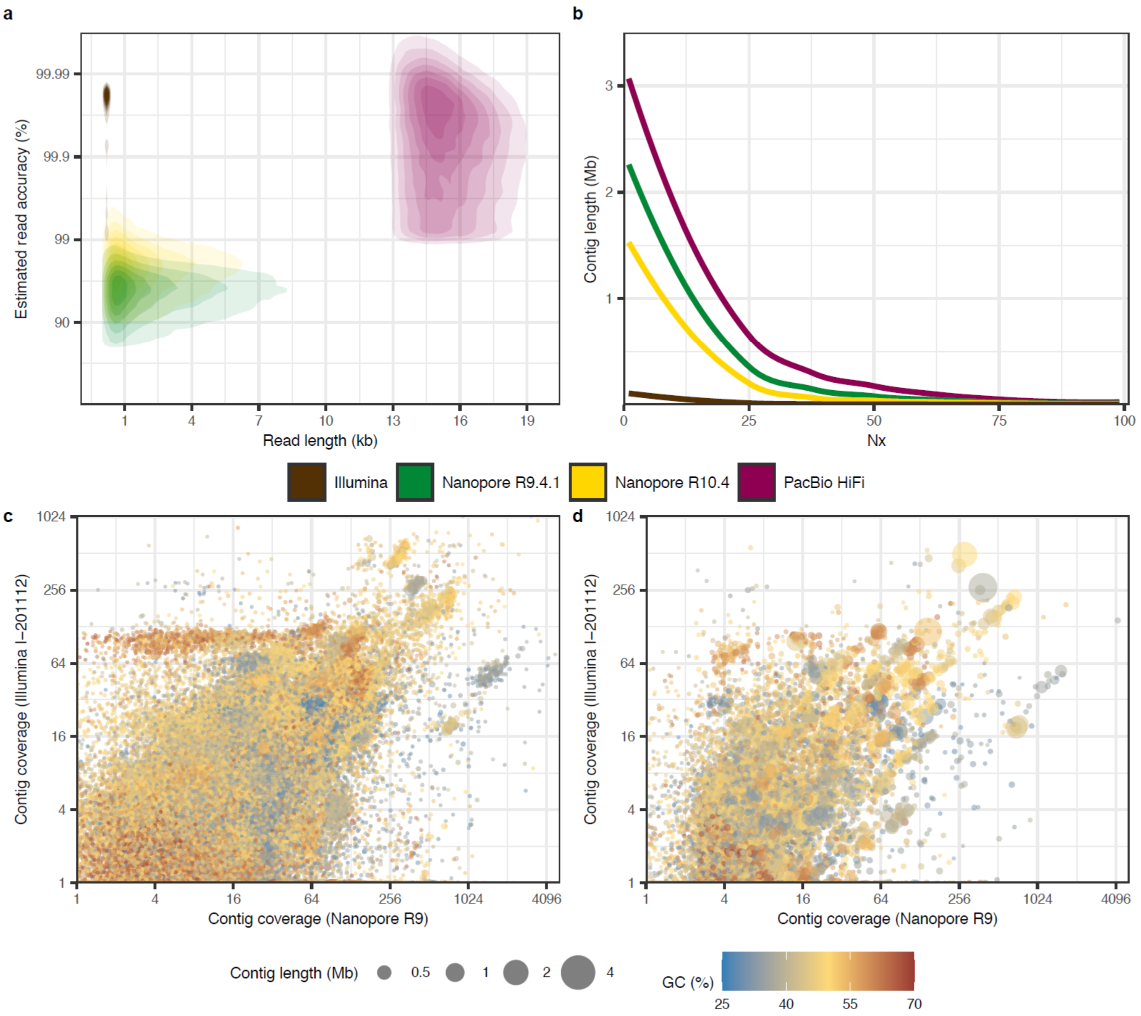

Figure S6: Sequencing and assembly overview for the anaerobic digester sample. **a)** Estimated read accuracy (from Q-scores) versus read length. Note that the PacBio HiFi sample underwent additional size selection prior to sequencing. **b)** Nx plot of the assemblies produced from different sequencing technologies. **c)** Differential coverage plot of the Illumina assembly. **d)** Differential coverage plot of the Nanopore R9.4.1 assembly.

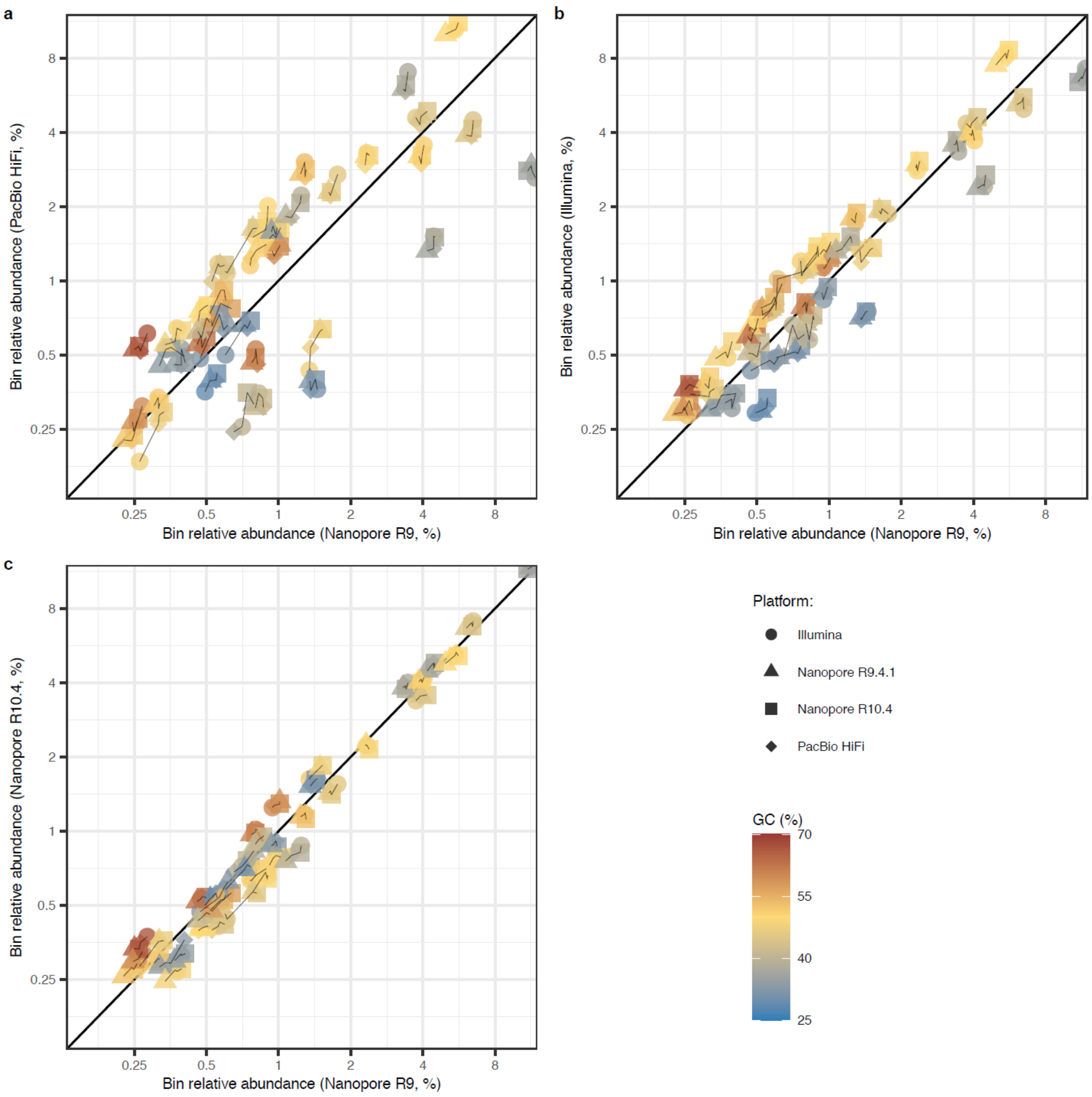

Figure S7: Comparison of bin relative abundances between different sequencing platforms. Relative abundance values (log-scaled) are presented between the Nanopore R9.4.1 data and **a)** PacBio HiFi, **b)** Illumina, **c)** Nanopore R10.4. Only the bins that were clustered together between different platforms are presented in the plots and are interlinked.

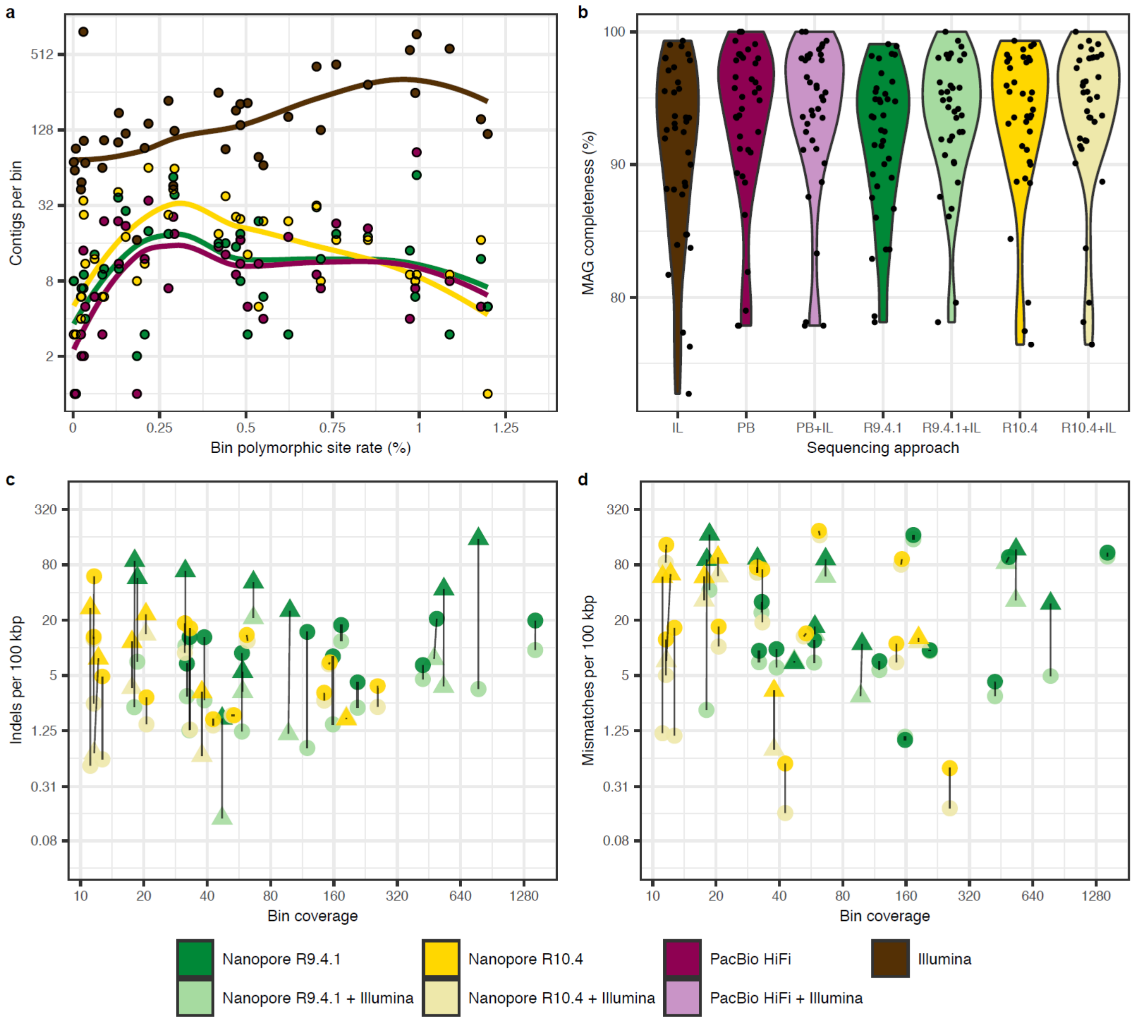

Figure S8: Comparison of bins from different sequencing approaches. **a)** MAG fragmentation (log-scaled) at different bin SNP rates in PacBio HiFi MAGs. Illumina-polished data was omitted from the plot, since short read polishing does not cause a change in contig counts. **b)** Genome bin completeness estimates for different sequencing platforms. IL: Illumina, PB: PacBio HiFi, R9.4.1: Nanopore R9.4.1, R10.4 : Nanopore R10.4. Bin **c)** indel and **d)** mismatch rates (log-scaled) for MAGs from Nanopore sequencing with and without Illumina read polishing, compared to MAGs from PacBio HiFi. The presented bin coverage on the x-axis (log-scaled) is for the corresponding Nanopore chemistry type. HQ MAGs are represented by circles, while triangles denote MQ MAGs. For all figures, only the bins that were clustered together between all the different sequencing platforms (hence, not all MAGs are included) are presented.

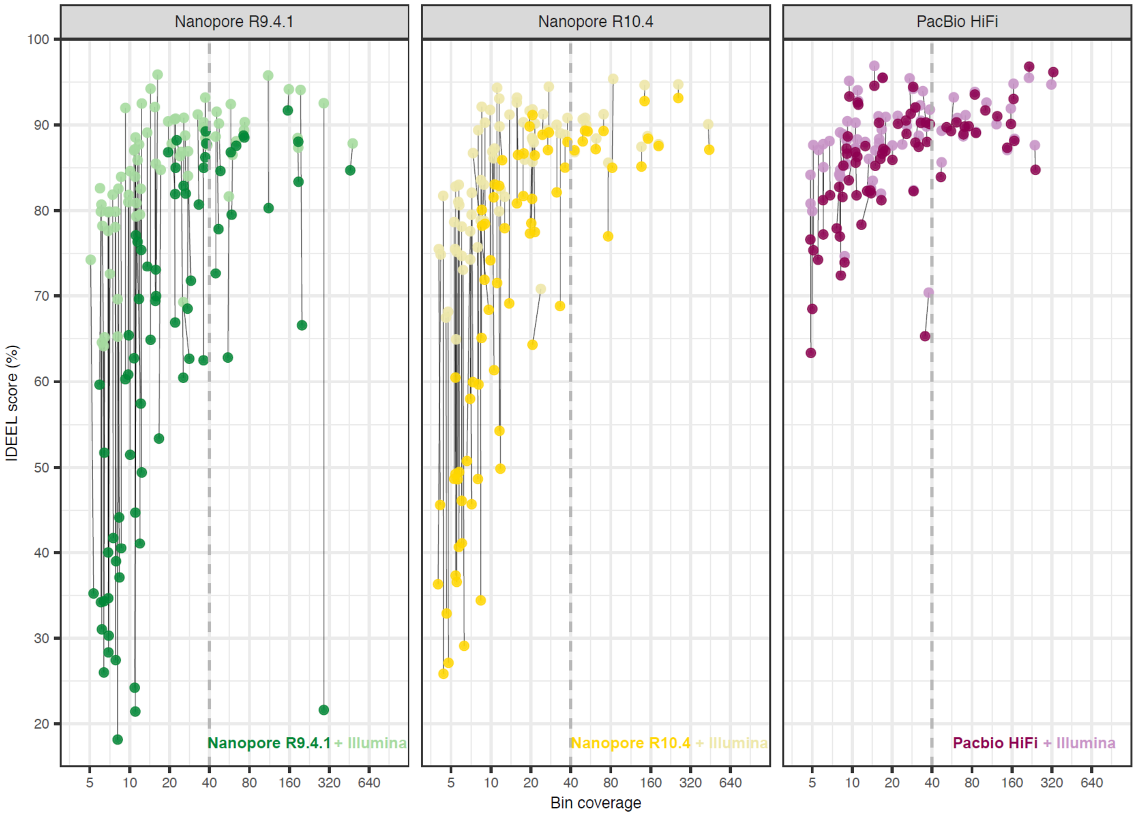

Figure S9: IDEEL score vs. coverage for metagenome bins from the anaerobic digester sample. The long-read bins are shown with and without Illumina polishing connected by a line. Only the bins that were clustered together between different long-read platforms (excluding Illumina-only bins, n=73 per platform) are presented in the plots. The plots feature 2 outliers. One is for the R9.4.1 MAG of 291x coverage, featuring an improvement in IDEEL score by 71, due to a very high count of long homopolymers (594 homopolymers of length 8 or above in the Illumina-polished MAG). The other outlier is a MAG that features reduced IDEEL scores across all datasets (21-38x coverage range, 63-71 IDEEL score). The MAG is classified as *Patescibacteria*, and only has distantly-related genomes in the database, which leads to systematically low IDEEL scores.

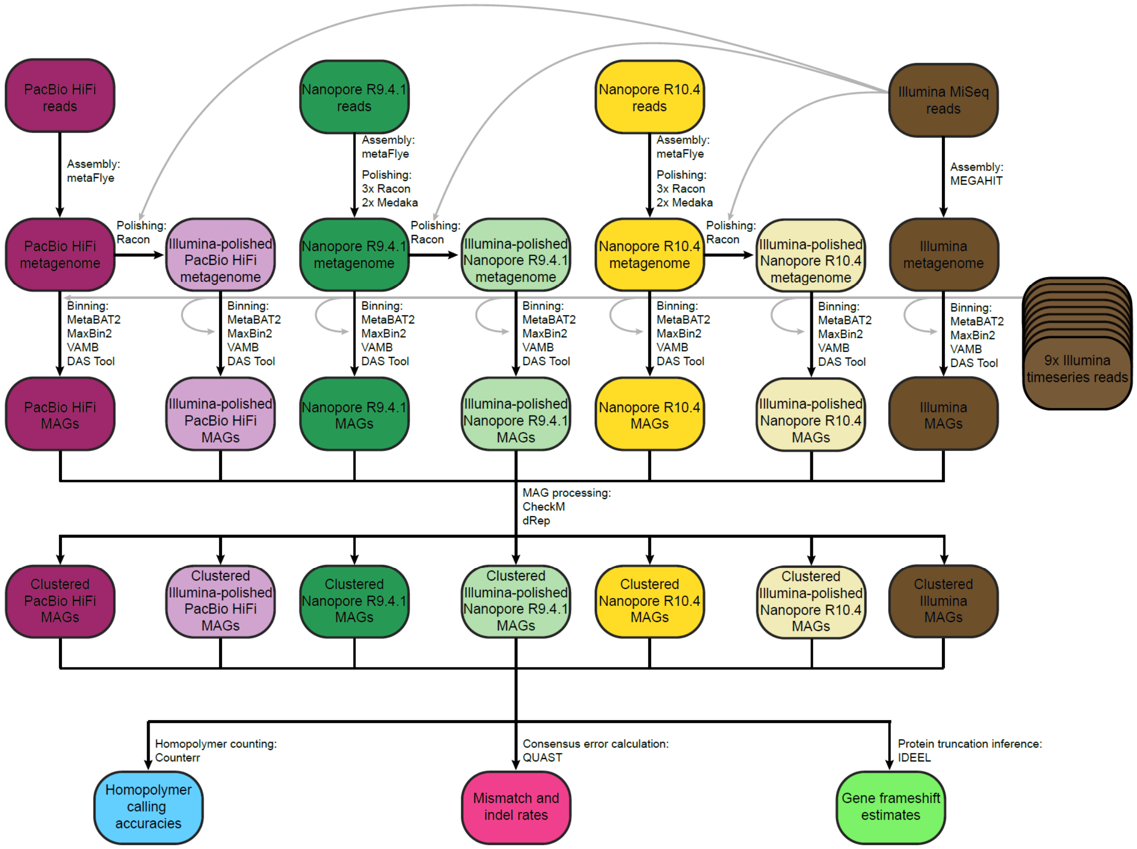

Figure S10: Schematic overview of the main bioinformatics processing steps with anaerobic digester datasets.

Table S1: Sequence statistics for the Zymo HMW Mock using different sequencing platforms. Estimated modal read accuracy is measured using the reported Q-score for each read type. Observed modal read accuracy was measured by read-mapping to the reference genomes. Slight variation in read quality between different Nanopore datasets can be caused due to technical variation of the sequencing experiments.

	**Illumina**	**Nanopore R9.4.1**	**Nanopore R10.4**
**Total read count**	48,123,500	8,846,993	22,452,567
**Total yield (Gb)**	7,2	31,6	52,3
**N50 (bp)**	151	14,018	5,992
**Estimated modal read accuracy (%)**	99.99	96.81	98.13
**Observed modal read accuracy (%)**	99.98	97.56	99.14

Table S2: Assembly and genome quality statistics for Zymo HMW mock bacterial species at 40x coverage. Note that the mismatches and indels might represent true diversity, as the available reference genomes from Zymo are not likely to be derived from the same batch as sequenced in this study.

Zymo species	Nanopore chemistry	Illumina polishing	Contigs	Assembly size (Mb)	Contig N50 (Mb)	Mismatches per 100kb	Indels per 100kb	ANI (%)	IDEEL score (%)
Bacillus subtilis	R9.4.1	N	1	4.0	4.0	0.42	2.74	99.995	96.28
		Y	1	4.0	4.0	0.40	1.09	99.998	98.46
	R10.4	N	1	4.0	4.0	0.42	1.29	99.998	98.54
		Y	1	4.0	4.0	0.42	1.29	99.998	98.54
Enterococcus faecalis	R9.4.1	N	1	2.8	2.8	2.78	7.59	99.993	97.42
		Y	1	2.8	2.8	2.50	1.27	99.998	99.17
	R10.4	N	1	2.8	2.8	2.53	1.30	99.998	99.32
		Y	1	2.8	2.8	2.50	1.16	99.998	99.32
Escherichia coli	R9.4.1	N	2	4.8	4.8	12.74	3.42	99.988	98.66
		Y	2	4.8	4.8	12.84	1.24	99.990	98.72
	R10.4	N	2	4.8	4.8	13.22	1.28	99.987	98.79
		Y	2	4.8	4.8	13.01	1.11	99.988	98.82
Listeria monocytogenes	R9.4.1	N	1	3.0	3.0	2.07	4.28	99.992	98.21
		Y	1	3.0	3.0	2.07	0.53	99.995	98.68
	R10.4	N	2	3.0	3.0	3.58	1.44	99.998	98.72
		Y	2	3.0	3.0	3.58	1.40	99.998	98.65
Pseudomonas aeruginosa	R9.4.1	N	1	6.8	6.8	9.28	2.28	99.991	99.09
		Y	1	6.8	6.8	9.29	0.38	99.993	99.19
	R10.4	N	1	6.8	6.8	9.70	0.32	99.993	99.23
		Y	1	6.8	6.8	5.26	0.24	99.993	99.23
Salmonella enterica	R9.4.1	N	2	4.8	4.8	0.46	2.23	99.996	96.14
		Y	2	4.8	4.8	0.44	0.40	99.998	96.31
	R10.4	N	4	4.8	4.8	1.09	0.59	99.996	96.24
		Y	4	4.8	4.8	0.63	0.48	99.997	96.27
Staphylococcus aureus	R9.4.1	N	1	2.7	2.7	0.18	2.61	99.994	98.72
		Y	1	2.7	2.7	0.18	0.74	99.996	98.78
	R10.4	N	1	2.7	2.7	0.33	1.18	99.999	98.61
		Y	1	2.7	2.7	0.18	0.66	99.999	98.74

Table S3: CMSeq SNP calling statistics for the Zymo HMW mock reference sequences using Illumina reads, indicating that some of the Zymo mock reference sequences include either assembly errors or real biological variation compared to the Lot's sequenced in this study.

	**Covered bases (Mb)**	**Polymorphic bases (bp)**	**Polymorphic rate**
Bacillus subtilis	4.0	10	2.5e-06
Enterococcus faecalis	2.8	113	4.0e-05
Escherichia coli	4.8	1156	2.4e-04
Listeria monocytogenes	3.0	80	2.7e-05
Pseudomonas aeruginosa	6.8	1222	1.8e-04
Salmonella enterica	4.8	41	8.6e-06
Staphylococcus aureus	2.7	18	6.6e-06

Table S4: Overview of read datasets used in the study.

Read dataset	Instrument	Yield (Gb)	Read N50 (kb)	Read count	ENA sample ID	LOT#
**IL-201104**	Illumina HiSeq	6.2	0.15	42,727,130	ERS7673063	
**IL-201112**	Illumina HiSeq	11.4	0.15	79,619,634	ERS7673064	
**IL-201301**	Illumina HiSeq	7.5	0.25	31,702,618	ERS7673065	
**IL-201308**	Illumina HiSeq	6.7	0.25	28,067,586	ERS7673066	
**IL-201502**	Illumina HiSeq	5.3	0.25	22,351,578	ERS7673067	
**IL-201702**	Illumina HiSeq	15.9	0.25	66,225,442	ERS7673068	
**IL-201705**	Illumina HiSeq	4.9	0.25	20,492,240	ERS7673069	
**IL-201707**	Illumina HiSeq	5.5	0.25	23,663,146	ERS7673070	
**IL-201804**	Illumina MiSeq	3.2	0.3	11,981,252	ERS7673071	
**IL-202001**	Illumina MiSeq	13.3	0.3	47,091,904	ERS7673072	
**PB-202001**	PacBio Sequel II	15.3	15.4	992,914	ERS7673073	
**R9-202001**	MinION Mk1B	35.2	5.9	10,266,261	ERS7673074	
**R10-202001**	MinION Mk1B	13.0	6.4	3,646,771	ERS7673075	
**R104-202001**	GridION	14.0	7.5	3,514,955	ERS7672969	
**IL-ZYMO**	Illumina MiSeq	7.5	0.15	49,774,986	ERS8296812	ZRC195845
**R941-ZYMO**	MinION Mk1B	32.0	1.8	8,851,918	ERS8296813	ZRC195845
**R104-ZYMO**	PromethION	5.2	7.5	18,831,686	ERS8296814	
