# Supplementary material for: Oxford Nanopore R10.4 long-read sequencing enables the generation of near-finished bacterial genomes from pure cultures and metagenomes without short-read or reference polishing
Source: Nat Methods. 2022 Jul 4;19(7):823–6. doi: 10.1038/s41592-022-01539-7 (PMC9262707; doi:10.1038/s41592-022-01539-7)
Supplement: Supplementary file 2 — Reporting Summary [file 41592_2022_1539_MOESM2_ESM.pdf]

nature research

Corresponding author(s): Mads Albertsen

Last updated by author(s): Apr 29, 2022

Reporting Summary

Nature Research wishes to improve the reproducibility of the work that we publish. This form provides structure for consistency and transparency in reporting. For further information on Nature Research policies, see our Editorial Policies and the Editorial Policy Checklist.

Statistics

For all statistical analyses, confirm that the following items are present in the figure legend, table legend, main text, or Methods section.

n/a	Confirmed	
☐	☒	The exact sample size (*n*) for each experimental group/condition, given as a discrete number and unit of measurement
☒	☐	A statement on whether measurements were taken from distinct samples or whether the same sample was measured repeatedly
☒	☐	The statistical test(s) used AND whether they are one- or two-sided<br>*Only common tests should be described solely by name; describe more complex techniques in the Methods section.*
☒	☐	A description of all covariates tested
☒	☐	A description of any assumptions or corrections, such as tests of normality and adjustment for multiple comparisons
☐	☒	A full description of the statistical parameters including central tendency (e.g. means) or other basic estimates (e.g. regression coefficient) AND variation (e.g. standard deviation) or associated estimates of uncertainty (e.g. confidence intervals)
☒	☐	For null hypothesis testing, the test statistic (e.g. *F*, *t*, *r*) with confidence intervals, effect sizes, degrees of freedom and *P* value noted<br>*Give P values as exact values whenever suitable.*
☒	☐	For Bayesian analysis, information on the choice of priors and Markov chain Monte Carlo settings
☒	☐	For hierarchical and complex designs, identification of the appropriate level for tests and full reporting of outcomes
☒	☐	Estimates of effect sizes (e.g. Cohen's *d*, Pearson's *r*), indicating how they were calculated

Our web collection on statistics for biologists contains articles on many of the points above.

Software and code

Policy information about availability of computer code

Data collection	MinKNOW software v21.05.25 (Oxford Nanopore, England) and Guppy v5.0.16 (Oxford Nanopore, England)
Data analysis	Cutadapt (v1.16), duplex-tools (v0.2.5, Oxford Nanopore), Porechop (v0.2.3), NanoFilt (v2.6.0), CCS (v6.0.0 Pacific Biosciences), NanoPlot (v1.24.0), Rasusa (v0.3.0), seqtk (v1.3), Counterr (v0.1), Flye (v2.9), Minimap2 (v2.17), Racon (v1.3.3), Medaka (v1.4.4, Oxford Nanopore), Megahit (v1.1.4), MetaBAT (v2.12.1), MaxBin2 (v2.2.7), Vamb (v3.0.2), DAS Tool (v1.1.2), CoverM (v0.6.1), CheckM (1.1.2), GTDB-tk (v1.5.0), tRNAscan-SE (v2.0.5), Prodigal (v2.6.3), Bowtie2 (v2.4.2), SAMtools (v1.9), CMseq (v1.0.3), dRep (v2.6.2), Diamond (v2.0.6), QUAST (v4.6.3), FastANI (v1.33), Barrnap (v0.9), Bioawk (v. 1.0), ncbi-genome-download (v0.3.0), infoseq (v. 6.6.0.0), https://github.com/Serka-M/Digester-MultiSequencing

For manuscripts utilizing custom algorithms or software that are central to the research but not yet described in published literature, software must be made available to editors and reviewers. We strongly encourage code deposition in a community repository (e.g. GitHub). See the Nature Research guidelines for submitting code & software for further information.

Data

Policy information about availability of data

All manuscripts must include a data availability statement. This statement should provide the following information, where applicable:

- Accession codes, unique identifiers, or web links for publicly available datasets
- A list of figures that have associated raw data
- A description of any restrictions on data availability

Raw sequencing reads are available on the European Nucleotide Archive: PRJEB48692 for the Zymo Mock microbial community and PRJEB48021 for the anaerobic digester sequencing data.
Bacterial genome assembly and additional files used in the study are available for download at https://doi.org/10.6084/m9.figshare.17008801.v1

UniProt TrEMBL database used in the study is available at https://ftp.uniprot.org/pub/databases/uniprot/previous_releases/release-2021_01/knowledgebase.
GTDB-tk database used in the study is available at https://data.ace.uq.edu.au/public/gtdb/data/releases/release202.
Zymo Mock community reference sequences are available at https://s3.amazonaws.com/zymo-files/BioPool/D6322.refseq.zip.
NCBI RefSeq genome database is available at https://ftp.ncbi.nlm.nih.gov/genomes/refseq.

Field-specific reporting

Please select the one below that is the best fit for your research. If you are not sure, read the appropriate sections before making your selection.

☒ Life sciences ☐ Behavioural & social sciences ☐ Ecological, evolutionary & environmental sciences

For a reference copy of the document with all sections, see nature.com/documents/nr-reporting-summary-flat.pdf

Life sciences study design

All studies must disclose on these points even when the disclosure is negative.

Sample size

We carried out sequencing of the Zymo Mock microbial community using three different sequencing strategies (ONT R9.4.1, ONT R10.4, Illumina MiSeq) to compare performance. The Zymo Mock was chosen as the composition of the community is know and the reference sequences are publicly available. For each sequencing strategy the Zymo Mock was sequenced at a depth providing at least 100x coverage for the bacterial species of the Zymo mock, which was deemed as sufficient for downstream analysis.

The single anaerobic digester sample was chosen as a proxy for a complex microbial community and was sequenced using four different sequencing strategies (ONT R9.4.1, ONT R10.4, Illumina MiSeq, PacBio HiFi) to compare performance. For each sequencing strategy, the anaerobic digester sample was sequenced at a minimal sequencing depth of 12 Gb, which, from previous projects, was expected to provide a sufficient amount of metagenome assembled genomes for downstream analysis.

Data exclusions

For comparing genome bins, bins which did not cluster between the different sequencing approaches, were excluded from direct comparisons. Also, long-read-based bins, which featured lower Illumina read coverage than 5, were excluded from direct comparisons. For the IDEEL test, query matches, which were not present in all datasets, were omitted to reduce noise. Also for the IDEEL test, the R.9.4.1 read dataset was sub-sampled to acquire bins at comparable coverage levels to other sequencing strategies.

Replication

For the Zymo Mock community, sequencing was performed independently. Zymo Mock bacterial genome assemblies were generated at multiple coverage levels to assess the impact of sequencing depth but also to assess the variability in genome quality metrics once adequate sequencing depth was achieved.

The anaerobic digester sludge sample was sequenced using 3 different sequencing methods (Illumina, PacBio CCS, Nanopore) and 2 different Nanopore chemistries (R9.4.1 and R10.4). Hence, the DNA sample from the anaerobic digester was sequenced 5 times (independently), but no biological replicates have been included in the study.

Sequencing of the 9 additional time-series Illumina datasets (no technical replicates) of the same anaerobic digester was performed independently from this study.

Randomization

Not relevant since this project does not use experimental groups

Blinding

Not relevant since this project does not use experimental groups

Reporting for specific materials, systems and methods

We require information from authors about some types of materials, experimental systems and methods used in many studies. Here, indicate whether each material, system or method listed is relevant to your study. If you are not sure if a list item applies to your research, read the appropriate section before selecting a response.

Materials & experimental systems

n/a	Involved in the study	
☒	☐	Antibodies
☒	☐	Eukaryotic cell lines
☒	☐	Palaeontology and archaeology
☒	☐	Animals and other organisms
☒	☐	Human research participants
☒	☐	Clinical data
☒	☐	Dual use research of concern

Methods

n/a	Involved in the study	
☒	☐	ChIP-seq
☒	☐	Flow cytometry
☒	☐	MRI-based neuroimaging
